# Research advances on the immune research and prospect of immunotherapy in pituitary adenomas

**DOI:** 10.1186/s12957-021-02272-9

**Published:** 2021-06-05

**Authors:** Ding Nie, Qiuyue Fang, Bin Li, Jianhua Cheng, Chuzhong Li, Songbai Gui, Yazhuo Zhang, Peng Zhao

**Affiliations:** 1 Department of Neurosurgery, Beijing Tiantan Hospital, Capital Medical University, Beijing, China; 2 Beijing Neurosurgical Institute, Beijing, China

## Abstract

**Background:**

Pituitary adenomas are one type of intracranial tumor, which can be divided into microadenoma (≤ 1 cm), macroadenoma (> 1 cm), and giant adenoma (≥ 4 cm) according to their diametral sizes. They are benign, typically slow-progressing, whereas the biological behavior of some of them is invasive, which presents a major clinical challenge. Treatment of some pituitary adenomas is still difficult due to drug resistance or multiple relapses, usually after surgery, medication, and radiation. At present, no clear prediction and treatment biomarkers have been found in pituitary adenomas and some of them do not cause clinical symptoms, so patients are often found to be ill through physical examination, and some are even found through autopsy. With the development of research on pituitary adenomas, the immune response has become a hot spot and may serve as a novel disease marker and therapeutic target.

The distribution and function of immune cells and their secreted molecules in pituitary adenomas are extremely complex. Researchers found that infiltration of immune cells may have a positive effect on the treatment and prognosis of pituitary adenomas. In this review, we summarized the advance of tumor immunity in pituitary adenomas, revealing the immunity molecules as potential biomarkers as well as therapeutic agents for pituitary adenomas.

**Conclusion:**

The immune studies related to pituitary adenomas may help us find relevant immune markers. At the same time, the exploration of immunotherapy also provides new options for the treatment of pituitary adenomas.

## Introduction

**Fig. 1:**
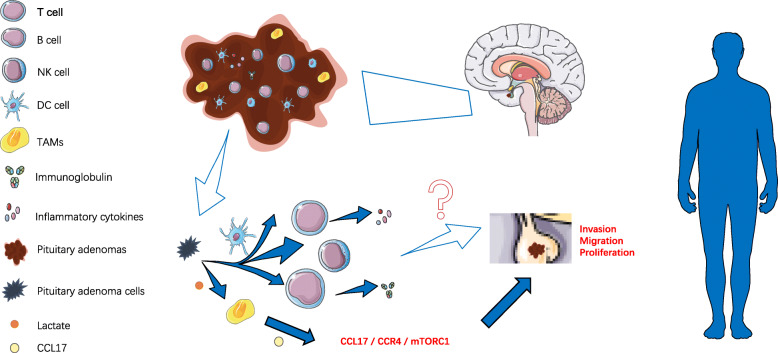
Immune cells infiltrate in pituitary adenoma. Immune cells infiltrate in PAs. The role of immune cells in PAs invasion, migration, and proliferation is unclear

Pituitary adenomas (PAs) are benign intracranial tumors with the third highest incidence, accounting for about 10–15% of intracranial tumors [1]. However, up to 20% of PAs appear clinically invasive symptoms, showing rapid growth and recurrence after treatment [2, 3]. More seriously, studies have shown that 0.2% of PAs are more likely to become pituitary cancer [4]. PAs can lead to clinical symptoms by oppressing the normal pituitary gland and invading the cavernous sinus or skull base structure. In addition, a variety of “functioning” PAs secrete supraphysiologic levels of hormones, resulting in profound systemic effects that reflect the changes in hormone levels [5]. At present, the treatment of PAs mainly depends on surgical resection [6]. However, the complete resection rate of tumors is only 66 to 78% [7]. When surgery and chemotherapy fail, radiotherapy becomes the treatment of choice against PAs. But radiation strikes at healthy tissue. For example, it can lead to visual impairment, hypopituitary, and cerebrospinal fluid leakage. Therefore, this clinical dilemma has inspired researchers to find new markers and treatment methods [8, 9]. With the deepening understanding of the tumor environment and its development, immunotherapy is a promising alternative therapy for the treatment of drug-resistant or recurrent PAs [9]. The mechanism of PAs occurrence and development is still unclear, which may be a result of multiple factors such as epigenetics, genes, and tumor microenvironment(TME) [10, 11]. The TME is a special environment generated by the interaction between tumor cells and the host during tumor development [12]. It is a complex environment consisting of fibroblasts, myofibroblasts, endothelial cells, immune cells, and extracellular matrix (ECM) which can affect tumor proliferation, invasiveness, and angiogenesis [13, 14]. Current research has focused on immune cells in TME. A tumor is a systemic disease in which inflammatory immune cells, chemokines, and cytokines influence tumor growth and invasion [15]. The infiltrating immune cells in brain tissue include macrophages, neutrophils, T cells, natural killer cells(NK cells), and other immune cells. Recent studies have shown that they play an important role in brain function and physiology; they can influence behavior and participate in the pathogenesis of various neuropathologies [16]. Myeloid cells, such as tumor-associated macrophages, dendritic cells, and lymphocytes, such as T cells and B cells, make up the tumor microenvironmental immune cells (TMICs) (Fig. 1) [17]. They may be located in the core, margin, or adjacent tertiary lymphoid structures (TLS) of the tumor [18]. The role of these infiltrating immune cells and their secreted molecules is complex and can ultimately lead to tumor-promoting or anti-tumor effects through interaction with the tumor and its host [19, 20]. There are still few studies on the immunity of pituitary adenoma. In this review, we summarize the current immunological studies on pituitary adenoma to point out the prospect that immunity molecules may be helpful to prognosis prediction and clinical targeted therapy.

## Immune cells infiltrate in PAs

Innate immunity (non-specific immunity) and adaptive immunity(specific immunity)comprise the human immune system, and adaptive immunity involves T and B cells and their secreted factors [21]. As part of the natural immune cell population, NK cells can control tumor growth by interacting with other immune cells and tumor cells [22, 23]. For example, MHC-I offer CD8^+^T lymphocytes cell antigens, such as from its protein and virus protein antigen, and extracellular antigen by MHC II usually provided to CD4^+^T lymphocyte, loss of MHC-I during the process of tumor formation is one typical method that cancer cells evade monitoring of CD8^+^T cells, NK cells express to identify MHC-I molecules-inhibition of cell surface receptors, and eliminate cannot fully express a large number of MHC-I molecular targets [24–26]. Current studies have shown that although the number of NK cells in solid tumors is smaller than that of CD8^+^T cells, CD4^+^T cells, and B cells, the presence of NK cells in TME may be associated with a good prognosis [27, 28]. Macrophages can be polarized into M1 or M2 macrophages. Tumor-associated macrophages(TAMs) are generally characterized as M2-like macrophages [29]. By stimulating tumor angiogenesis and inhibiting the anti-tumor immune response mediated by T cells, TAMs promote the proliferation, invasion, and metastasis of tumor cells [30]. Recent studies by Zhang et al. have shown that PA may produce excess lactate, resulting in TME acidification, which reshapes TAMs into an M2-type phenotype and then secrets CCL17 through TAMs to enhance tumor invasion through the CCL17/CCR4/mTORC1 axis [31]. Meanwhile, M2 macrophages may play a role in neoangiogenesis in pituitary adenomas together with B cells, CD4 ^+^T cells, and Foxp3 ^+^ lymphocytes [32]. Dendritic cells (DCs) are the main antigen-presenting cells and serve as a bridge between adaptive and innate immune systems [33]. DCs promotes tumorigenesis in some tumors and inhibits tumorigenesis in others, which indicates the phenomenon of tumor stage dependence, that is, DCs show tumor inhibition effect in the early stage, but with the tumor promotion, it can be changed to promote the development of the tumor [33]. A key part of the adaptive immune response is CD4^+^T and CD8^+^T cells [34, 35]. Current studies have shown that CD4^+^T cells mediate anti-tumor responses through a variety of mechanisms, such as the CCR5 ligand that acts as the center of CD4^+^ and CD8^+^T cell activation. CD8^+^T cells destroy target cells by differentiating into cytotoxic T cells (CTLS), releasing cytotoxic particles [36]. In addition, B cell subsets have immunosuppressive and/or regulatory functions and may play a key role in regulating the human immune response to tumors [37].

Research conducted by Wang et al. showed that there is a different distribution of tumor-infiltration immune cells (TIICs) between PAs and the normal pituitary gland, and there are also differences among different PA subtypes. In addition, three immune clusters can be identified among PAs according to their distribution, and each one of PAs shows unique characteristics [38]. PAs can be classified into functional pituitary adenomas (FPAs) and non-functional pituitary adenomas (NFPAs) according to the hormone secretion [39]. FPAs include prolactinomas, somatotroph, corticotroph, thyrotrophin, and rarely gonadotroph adenomas [40]. Existing studies have shown that immune cells infiltrate differently in both FPAs and NFPAs [14, 41–43]. According to imaging characteristics and pathological diagnosis, PAs can be divided into invasive PAs and non-invasive PAs [44, 45]. There are also inconsistencies in the distribution of infiltrating immune cells in PAs, depending on the aggressiveness [46, 47]. For example, the infiltration of CD8^+^T cells was positively correlated with PRL and PRL and GH immunostaining in the tumor and was also positively correlated with the invasions of PAs [41, 48]. The research of recent years has been recently reviewed by us and is presented in Table 1 [5, 14, 32, 41, 42, 47–59]. It is worth noting that the role of immune cells in PAs is not yet clear and may vary among different tumor stages and types. Therefore, before the exact role of immune cells in pituitary tumors is understood, it is necessary to define the immune characteristics of PAs [59].

**Table 1:** Immune studies of PAs

Cell type and marker	Conclusion/distribution	Ref.	Population	Methods	Year
CD3 (T cells)	FPAs > NFPAs; GH adenomas > ACTH adenomas > PRL adenomas.	[5, 49]	67; 48	Immunohistochemistry	2020; 2016
CD4 (T cells)	FPAs > NFPAs; GH adenomas > non-GH adenomas. T cell phenotype was the CD4^+^ memory resting phenotype. May have angiogenic effects.	[32, 41, 48, 49]	24; 134; 35; 48	Immunohistochemistry	2020 ;2020; 2015; 2016
CD8 (T cells)	GH adenomas > non-GH adenomas. Positively correlated with serum PRL and intratumoral immunostaining of PRL and GH. Cavernous sinus invasion > non-invasive tumors. The number of CD8^+^ lymphocytes was positively correlated with the number of CD68^+^ macrophages. FPAs > NFPAs (especially GHomas). Cushing pituitary tumors had higher CD8^+^ T cells.	[41, 42, 48, 50–52]	134; 191; 35; 27; 64; 115	Immunohistochemistry; Computational approach; RNA-seq.;	2020; 2018; 2015; 2019; 2020; 2020
FOXP3 (regulatory T cells)	AIP-mutated GH tumors > sporadic ones and NPG. Cavernous sinus invasion > non-invasive tumors. May have angiogenic effects.	[32, 50, 53]	24; 27; 15	Immunohistochemistry	2020; 2019; 2019
CD20 (B cells)	FPAs > NFPAs (especially GHomas). May have angiogenic effects.	[32, 52]	24; 115	Immunohistochemistry; RNA-seq.	2020; 2020
CD45 (lymphocytes)	The CD45 staining in pituitary adenomas was significantly greater than that in normal pituitary. There was no statistically significant difference among the various secretory types. High (MIB-1 > 3%) proliferative indices > low (MIB-1 ≤ 3%) proliferative indices.	[49, 54]	48; 72	Immunohistochemistry	2016; 2010
CD68 (macrophages)	The numbers of CD68^+^ cells showed a positive correlation with the tumor sizes and Knosp classification grades. Sparsely granulated GH and null cell tumors > densely granulated GH and ACTH tumors. AIP-mutated GH tumors > sporadic ones and NPG. The number of CD8^+^ lymphocytes was positively correlated with the number of CD68^+^ macrophages. Gonadotroph PitNETs present an increased CD68^+^ macrophage signature compared to somatotroph, lactotroph, and corticotroph PitNETs. The percentage of CD68^+^ and CD163^+^ infiltrating macrophages was significantly associated with the aggressiveness of gonadotropin tumors. Macrophages and NK cells are positively correlated. M2 macrophages > M1 macrophages. In the PA stroma, CD68+ macrophages > CD4+ T cells and CD8+ T cells.	[31, 41, 47, 48, 51–53]	35; 134; 28;35; 64; 115; 15	Immunohistochemistry; Computational approach; flow cytometry	2021; 2020; 2020; 2015; 2020; 2020; 2019
CD147	Invasion tumors > non-invasive tumors.	[55]	55	Immunohistochemistry	2005
CD163	The most abundant type of immune cell in PitNETs, and mainly CD163 +.	[14]	45	immunohistochemistry	2019
NK cells	Macrophages and NK cells are positively correlated.	[52]	115	Immunohistochemistry; RNA-seq.	2020
Neutrophils	PitNETs contained fewer neutrophils. NF-PitNETs had more neutrophils than somatotropinomas.	[14]	45	Immunohistochemistry	2019
CTLA-4	There was no significant difference in CTLA-4 expression among tumor subtypes.	[52]	115	Immunohistochemistry; RNA-seq.	2020
PD-1	NFPAs>FPAs (especially GHomas). High (MIB-1 > 3%) proliferative indices > low (MIB-1 ≤ 3%) proliferative indices.	[49, 52]	48; 115	Immunohistochemistry	2016; 2020
PD-L1	FPAs > NFPAs (especially GHomas). Positively correlated with serum PRL and intratumoral immunostaining of PRL and GH. The score tended to be higher (*p* = 0.050) in the cavernous sinus invasion group. There was no difference between primary and recurrent adenomas.	[5, 42, 49, 50, 52, 56]	67; 191; 48; 27; 115; 55.	Immunohistochemistry; RNA-seq.	2020; 2018; 2016; 2019; 2020; 2020

*Abbreviations*: *FPAs* functional pituitary adenoma, *NFPAs* non-functional pituitary adenoma, *GH* growth hormone, *PRL* prolactin, *ACTH* adrenocorticotropic hormone, *FoxP3* forkhead box protein P3, *NK cell* natural killer cell, *PitNETs* pituitary neuroendocrine tumor, *NPG* normal pituitary glands, *AIP* aryl hydrocarbon receptor-interacting protein, *CTLA-4* co-inhibitory cytotoxic T lymphocyte-associated protein 4

## Inflammatory factors associated with PAs

Current research has shown that cytokines including interferon (IFN), interleukin insurance-linked securities (ILs), and tumor necrosis factor (TNF) play a key role in the differentiation of the pituitary gland and oncogenesis of PAs [60, 61]. T helper type 1 (Th1) cells secrete TNF-α, IFN-γ, and IL-2, and they induce cell-mediated immune responses, whereas Th2 cells secrete IL-4, IL-5, IL-6, IL-10, and IL-13 [60]. IFN is divided into type I and type II. The main classes of type I IFN are IFN-α, IFN-β, IFN-ɛ, IFN-κ, and IFN-ω. IFN-γ belongs to type II IFN [62]. IFNs play a regulatory role in pituitary hormone secretion. Both stimulatory and inhibitory effects of IFNs (IFNα and IFNγ) on the secretion of ACTH, PRL, and GH have been reported [63]. IFN-α significantly inhibits hormone secretion and intracellular hormone concentration in human GH secreting PAs, prolactin, and NFPAs or gonadotropin adenoma [63]. TNF can trigger a variety of potential outcomes through TNFR1 and TNFR2 activation signals, including cell proliferation, gene activation, or cell death [64]. In invasive PAs, TNF-α promotes pathological osteoclast formation by directly inducing osteoclast differentiation, leading to inflammatory bone destruction [65]. Therefore, Zhu et al. proposed that TNF-α might be a novel target in the treatment of osteo-invasive pituitary adenoma [66]. TNF-α also may be an important regulator of hemorrhagic transformation in pituitary adenomas indicated by the finding that hemorrhagic pituitary adenomas displayed higher protein and mRNA levels of TNF-α [67]. The function of ILs is related to the expression and regulation of the immune response, which is involved in many factors originating from lymphocytes or macrophages. Qiu et al. reported that in the serum of patients with PAs (include invasive and non-invasive PAs), IL-4, IL-5, and IL-17A were significantly increased, while Th1/Th2 ratio was significantly decreased [60]. Notably, serum IL-17A levels in patients with invasive PA reported in this study were significantly higher than those in patients with non-invasive PA. While another study showed no significant difference between serum IL-17A levels and PA growth types [68]. In addition, the serum level of IL-4 was significantly higher in patients with idiopathic hyperprolactinemia [69]. The bidirectional role of IL-6 in PAs has also been reported. Paracrine IL-6 may be a condition that permits the growth of pituitary cells by contributes to excessive hormone production, growth, and neovascularization of pituitary adenomas, while autocrine IL-6 inhibits the aggressive growth and malignant transformation of tumors [70]. In vitro studies, an earlier study showed that IL-2 and IL-6 stimulated the proliferation of GH3 cells [71]. Furthermore, other inflammatory factors present in TME also play complex roles that require further detailed exploration by researchers. For example, macrophage migration inhibitory factor (MIF) is an immunomodulator that can be induced by pituitary hormones, enhancing the production of inflammatory cytokines such as TNF, IL-1, and IFN, to play its role of anti-tumor or tumor promotion [72].

## Status of immunotherapy for PAs

The relationship between tumor cells and the immune system is divided into three stages according to the theory proposed by Jun et al., at the initial stage, tumor cells are recognized and cleared by immune cells. As tumors develop, there is a phase of balance between tumor cells and immune cells. Eventually, the immune response is evaded by the tumor cells, and the immune system is unable to cope with the tumors [73]. As the tumor grows and changes, tumor cells can evade the immune system, leading to further spread, infiltration, and even metastasis. This kind of avoidance is achieved by the co-stimulation and co-inhibition signals of tumor cells [74]. The immune checkpoint is a regulator of immune activation and plays an important role in maintaining self-tolerance, controlling immune response intensity, and reducing tissue damage [75]. Immune checkpoints can be used by tumors to suppress T cell activation. The most representative ones are the co-inhibitory cytotoxic T lymphocyte-associated protein 4 (CTLA-4) and programmed cell death 1 (PD-1) pathways. T cell dysfunction, failure, and neutralization in tumors can be caused by activation of the PD-1 signaling pathway [76]. CTLA-4 is expressed in various types of tumors including PAs and can play a role by limiting the CD4^+^T cell phenotype [77, 78]. As a result, blocking immune checkpoints is a new approach to the treatment of many types of tumors [79]. PD-L1 binds to the PD-1 receptor on activated T cells and inhibits the cytotoxic anti-tumor function of T cells while blocking this interaction can produce a lasting T cell response [80–82]. Blocking CTLA-4 receptors on lymphocytes leads to T cell activation, which reduces tumor-mediated immune tolerance [83]. When the CTLA-4 and PD-1 are blocked, the stimulation signal of T cells are activated, the number of cytotoxic T cells with anti-tumor activity increase, the production and proliferation of pro-inflammatory cytokines can also be promoted, and finally, the tumor destruction can be accelerated [84, 85]. While anti-programmed cell death protein 1 (anti-PD-1) and anti-cytotoxic T-lymphocyte-associated protein 4 (anti-CTLA4) antibodies have been extensively used to target immune checkpoints in many cancers, their use in pituitary tumors has just commenced.

Recently, it has been reported that PD-L1 expression is higher in pituitary tumors invading the cavernous sinus [86]. In addition, the expression of PD-L1 also significantly increased with the increase of serum GH, PRL, ACTH, and cortisol levels [42]. Similar conditions also exist in the expression of PD-1 [49, 52]. However, no significant difference was found in CTLA4 expression [52]. In animal experiments, Hanna R et al. provided a new theoretical basis for the immunotherapy of PAs, that is, anti-PD-L1 treatment successfully reduced the plasma ACTH level of model mice, reduced the growth of PAs, and improved the survival rate of model mice [5]. In clinical application, a patient with ACTH-secreting pituitary carcinoma who received clinical remission using ipilimumab (anti-CTLA-4) and nivolumab (anti-PD-1) ICI was reported by Sol et al. [87]. Moreover, immune checkpoints such as TIM3 and LAG3 are also potential targets for immunotherapy of pituitary adenomas [88]. In addition to immune checkpoints, other methods of immunotherapy are being explored. Hazrati et al. published a report of a female with a macroprolactinoma refractory to conventional therapy and was successfully treated with immunotherapy. A Th1 activator adjuvant was inoculated with autoantigens weekly for 24 weeks, and following this therapy, her serum prolactin levels decreased and adenoma almost disappeared [89]. However, the side effects of immunotherapy are varied and any organ (skin, intestines, liver, and glands like the thyroid and adrenal glands) may be affected, leading to a variety of diseases (rash, pruritus, vitiligo, diarrhea, colitis, hepatitis, hypophysitis, hypothyroidism, primary adrenal insufficiency, and diabetes) [90, 91]. So, further studies are needed to refine the immunotherapy regimen for PAs. For example, immunotherapy combined with radiation therapy may be a promising option [92].

## Future perspectives and conclusions

At present, the specific mechanism of the interaction between pituitary adenoma and the human immune system is not clear, and studies are limited to the apparent expression level of related markers. However, based on the existing evidence, namely, the expression of immune cells and the changes and differences of inflammatory factors in pituitary adenomas, the immune research on pituitary adenomas deserves further study. The following points may be the direction of efforts: (1) comprehensive analysis of the expression of different immune cells and immune factors in the same batch of samples. (2) The study should be conducted in different types of pituitary adenomas. (3) Further mechanism study based on the current apparent expression research. (4) Exploration of new immune cell types and immune factors. At the same time, the exploration of immunotherapy also provides a new choice for the treatment of PAs. It should also be kept in mind that the immune response is dynamic during tumor development and that there may be inter-patient heterogeneity. Individualized immunotherapy strategies for individual patients at different stages are very important. Therefore, it is necessary to determine the immune landscape and its mechanism changes in pituitary adenoma, which is undoubtedly a challenge for researchers.

## Data Availability

Not applicable.

## Abbreviations

PAs: Pituitary adenomas
TME: Tumor microenvironment
NK: Natural killer
ECM: Extracellular matrix
TMICs: Tumor microenvironmental immune cells
TLS: Tertiary lymphoid structures
TAM: Macrophages
DCs: Dendritic cells
CTLS: Cytotoxic T cells
FPAs: Functional pituitary adenomas
NFPAs: Non-functional pituitary adenomas
TIICs: Tumor-infiltration immune cells
IFN: Interferon
ILs: Interleukin insurance-linked securities
TNF: Tumor necrosis factor
MIF: Macrophage migration inhibitory factor
CTLA-4: Co-inhibitory cytotoxic T lymphocyte-associated protein 4
PD-1: Programmed cell death 1
